# The Protective Role of Antioxidants in the Defence against ROS/RNS-Mediated Environmental Pollution

**DOI:** 10.1155/2014/671539

**Published:** 2014-07-20

**Authors:** Borut Poljšak, Rok Fink

**Affiliations:** Faculty of Health Sciences, University of Ljubljana, Zdravstvena pot 5, SI-1000 Ljubljana, Slovenia

## Abstract

Overproduction of reactive oxygen and nitrogen species can result from exposure to environmental pollutants, such as ionising and nonionising radiation, ultraviolet radiation, elevated concentrations of ozone, nitrogen oxides, sulphur dioxide, cigarette smoke, asbestos, particulate matter, pesticides, dioxins and furans, polycyclic aromatic hydrocarbons, and many other compounds present in the environment. It appears that increased oxidative/nitrosative stress is often neglected mechanism by which environmental pollutants affect human health. Oxidation of and oxidative damage to cellular components and biomolecules have been suggested to be involved in the aetiology of several chronic diseases, including cancer, cardiovascular disease, cataracts, age-related macular degeneration, and aging. Several studies have demonstrated that the human body can alleviate oxidative stress using exogenous antioxidants. However, not all dietary antioxidant supplements display protective effects, for example, *β*-carotene for lung cancer prevention in smokers or tocopherols for photooxidative stress. In this review, we explore the increases in oxidative stress caused by exposure to environmental pollutants and the protective effects of antioxidants.

## 1. Introduction

Many environmental pollutants are sources of several reactive species (RS). RS is a collective term that includes both oxygen radicals and other reactive oxygen and nitrogen species (ROS/RNS). Free radicals, important for living organisms, include hydroxyl (OH^•^), superoxide (O_2_^•−^), nitric oxide (NO^•^), thyl (RS^•^), and peroxyl (RO_2_^•^) radical. Peroxynitrite (ONOO^−^), hypochlorous acid (HOCl), hydrogen peroxide (H_2_O_2_), singlet oxygen (^1^O_2_), and ozone (O_3_) are not free radicals but can easily lead to free radical reactions in living organisms. The term reactive oxygen species (ROS) is often used to include not only free radicals but also the nonradicals (e.g., ^1^O_2_, ONOO^−^, H_2_O_2_, O_3_) [1].

There is strong evidence that RS is involved in oxidative/nitrosative stress (O/NS) as a common mechanism by which several environmental pollutants induce damage. Oxidative stress can be defined as an excessive amount of RS, which is the net result of an imbalance between production and destruction of RS (the latter is regulated by antioxidant defences). Oxidative stress is a consequence of an increased generation of RS and/or reduced physiological activity of antioxidant defences against RS. Environmental pollutants stimulate a variety of mechanisms of toxicity on molecular level and oxidative stress seems to be the common denominator leading to the damage to cellular membrane lipids, DNA, and proteins [2], as well as modulation of antioxidant enzymes. RS are, due to their high reactivity (e.g., hydroxyl radical formation), prone to cause damage to any type of molecule within the cell, for example, polyunsaturated fatty acids, glutathione, certain amino acids, and so forth.

When the antioxidant defence in the human body becomes overwhelmed, oxidative stress to the cellular components often occurs, inducing inflammatory, adaptive, injurious, and reparative processes [3]. On other hand, lifestyle and nutrition might play an important role against environmental oxidant exposure and damage. Protection against O/NS-mediated environmental pollutants can generally occur at two levels: (i) physiochemical protection to lower the dose of exposure, which typically cannot be accomplished by individuals living in polluted areas, or (ii) physiological protection to increase the antioxidative defence of the organism. There is growing scientific evidence that low molecular-weight antioxidants are involved in the prevention of or the decrease in the damage caused by certain environmental pollutants. Because we have little influence on the increasing levels of endogenous antioxidants, it would be reasonable to increase the amount of exogenous antioxidants (mainly through ingestion) to strengthen the defensive properties of organisms against environmental oxidative stress. The current evidence suggests that increased consumption of fruits and vegetables or certain dietary supplements can substantially enhance the protection against many common types of environmentally induced O/NS.

## 2. Purpose

This review aims to determine whether antioxidants can modulate the toxicity of environmental pollutants, thereby influencing health and disease outcome associated with oxidative stress-induced insults. Evidence will be presented that environmental pollution increases oxidative stress and that dietary supplementation with antioxidants may play a role on the neutralization or buffering of the effects of pollutants with oxidizing properties. The recommendation for the use of dietary antioxidants in areas of increased environmental pollution will be discussed.

This review summarises the most common and health-relevant sources of oxidative stress like air pollution, radiation, pesticides, noise, and household chemicals. Due to space constrains and a broad scientific data, not all the studies could be covered in this review. The reader is thus referred to search through provided references (and references therein) for further details on selected environmental pollutant or selected antioxidant.

### 2.1. Air Pollution-Induced Oxidative Stress and Protection against It

The health effects of air pollution result from minor irritation of the eyes and the upper respiratory system to chronic respiratory disease, heart and vascular disease, lung cancer, and death. Different studies presented in Table 1 are demonstrating increased oxidative stress/damage due to air pollutant exposure and that antioxidants could offer certain level of protection [4–7].

Oxygen could be presented as the leading air pollutant in regard to oxidative stress formation. Molecular O_2_ itself qualifies as a free radical because it has two unpaired electrons with parallel spin in different *π*-antibonding orbitals. This spin restriction accounts for its relative stability and paramagnetic properties. O_2_ is capable of accepting electrons to its antibonding orbitals, becoming “reduced” in the process, and, therefore, functioning as a strong oxidizing agent [76]. The diatomic molecule of oxygen contains two uncoupled electrons and can therefore undergo reduction, yielding several different oxygen metabolites, which are collectively called ROS. Mitochondria are the main site of intracellular oxygen consumption and the main source of ROS formation [8, 10, 13, 77]. Once ROS are produced, they are removed by cellular defenses which include the enzymes superoxide dismutase (Mn-SOD, Cu/Zn-SOD, and extracellular (EC)-SOD), catalase, glutathione peroxidase, peroxiredoxins, and the nonenzymatic antioxidants, like glutathione (GSH), thioredoxin, ascorbate, *α*-tocopherol, and uric acid [9, 78]. Since oxidative damage of cells increases with age, the increased intake of exogenous antioxidants may support the endogenous antioxidative defense. Clinical studies imply that eating a diet rich in fruits, vegetables, whole grains, legumes, and omega-3 fatty acids can help humans in decreasing oxidative stress and postponing the incidence of degenerative diseases [79].

Ozone is formed from dioxygen by the action of ultraviolet light and atmospheric electrical discharges. Ozone is a very reactive gas whose uptake depends on the availability of antioxidants in the lining fluids [17, 18, 52]. The surface of the lung is covered with a thin layer of fluid that contains a range of antioxidants that appear to provide the first line of defence against air pollutants. Mudway et al. [17] studied the interaction of ozone with antioxidants and found that the hierarchy toward ozone in human epithelial lining fluid was ascorbic acid followed by uric acid and then glutathione. Wu and Meng [34] analysed the effects of sea buckthorn seed oil on the protection against sulphur dioxide inhalation. They found that buckthorn seed oil contributed antioxidant effects. Furthermore, study by Zhao et al. [33] revealed the protective effect of salicylic acid and vitamin C on sulphur dioxide-induced lipid peroxidation in mice.

Tobacco smoke is one of the most common air pollutants and generates high amounts of various ROS/RNS. Cigarette-induced oxidative stress was found to be affected by the protective effects of vitamin C, glutathione, and other antioxidants, mainly as quenchers of ROS/RNS (Table 1) [36–41].

Kienast et al. [54] demonstrated that alveolar macrophages and peripheral blood mononuclear cells become activated following exposure to nitrogen dioxide. Several studies have demonstrated that certain antioxidants might play a beneficial role in NO_*x*_-induced toxicity. Guth and Mavis [55] and Sevanian et al. [56, 80] examined the effect of vitamin E content on the lungs. Furthermore, a study by Böhm et al. [62] revealed that dietary uptake of tomato lycopene protects human cells against nitrogen dioxide-mediated damage. The possible influence of dietary antioxidants, especially vitamin C, on the increasing prevalence of asthma was explored by Hatch [81].

Particulate matter can also cause oxidative stress via direct generation of ROS from the surfaces of soluble compounds, altering the function of mitochondria or reducing the activity of nicotinamide adenine dinucleotide phosphate-oxidase, inducing the activation of inflammatory cells to generate ROS and RNS and mediating oxidative DNA damage [63, 82]. Antioxidants could also provide protection against particulate matter-induced toxicity. Indeed, lung lining fluid antioxidants (urate, glutathione, and ascorbate) were demonstrated to be effective in a study by Greenwell et al. [83]. Luo et al. [70] detected an inhibitory effect of green tea extract on the carcinogenesis induced by the combination of asbestos and benzo(a)pyrene in rats drinking 2% green tea extract throughout their lives.

As the diet is the main source of antioxidant micronutrients, a plausible link now exists between the exposure to air pollution and the quality of food consumed.

### 2.2. Radiation-Induced Oxidative Stress and Protection against It

Ionising radiation consists of highly energetic particles which can generate ROS. These ROS can either be generated primarily via radiolysis of water or they may be formed by secondary reactions. Extensive doses of ionizing radiation have been shown to have a mutating effect; for example, Sperati et al. [84] concluded that indoor radioactivity appears to affect the urinary excretion of 8-OHdG among females, who are estimated to exhibit a higher occupancy in the dwellings measured than males (Table 2). Many compounds have been demonstrated to protect against cell injury caused by radiation-induced ROS formation. One of these compounds is ebselen, a selenoorganic compound [85]. Another compound is N-acetylcysteine, which reduces nitrosative damage during radiotherapy [86] as well as oxidative damage [87]. The radioprotective effects of quercetin and the ethanolic extract of propolis in gamma-irradiated mice were also detected [88]. The radioprotective and radiosensitising activities of curcumin were demonstrated in a study by Jagetia [89].

Aside from ionising radiation, nonionising radiation also causes oxidative stress. Magnetic fields can affect biological systems by increasing the release of free radicals. There are several studies that indicate a relationship between electromagnetic fields, ROS levels, and OS to exert toxic effects on living organisms [90]. Because it is unlikely that electromagnetic fields can induce DNA damage directly due to their low energy levels, most studies have examined their effects on the cell membrane, general and specific gene expression levels, and signal transduction pathways [91]. Musaev et al. [92] indicated that decimetric microwaves exert oxidant effects at a high intensity of irradiation (specific absorption rate of 15 mW/kg) and antioxidant effects at a low intensity (specific absorption rate of 5 mW/kg) (Table 2). The protective effects of melatonin and caffeic acid phenethyl ester against retinal oxidative stress during the long-term use of mobile phones were reported [93]. Jajte et al. [94] concluded that melatonin provides protection against DNA damage to rat lymphocytes. Another investigation revealed that* Ginkgo biloba* prevents mobile phone-induced oxidative stress [95]. Guney et al. [96] found that vitamins E and C reduce phone-induced endometrial damage.

Visible and UV light are insufficient to ionize most biomolecules. Nevertheless, human exposure to ultraviolet radiation has important public health implications. Although the skin possesses extremely efficient antioxidant activities, during aging, the ROS levels rise and the antioxidant activities decline. In addition, UV exposure to the skin results in the generation of ROS [118], such as singlet oxygen, peroxy radicals, the superoxide anion, and hydroxyl radicals, which damage DNA and non-DNA cellular targets [113–116] and accelerate the skin aging process. UV-radiation alters endogenous antioxidant protection; for example, in a study by Shindo et al. [127], after UV-irradiation, the epidermal and dermal catalase and superoxide dismutase activities were greatly decreased. With respect to the protective role of antioxidants, many studies (Table 2) investigated the effect of vitamin C on ultraviolet-radiation- (UVR-) induced damage. Oral vitamin C supplements resulted in significant increases in plasma and skin vitamin C content [118]. In the study by Aust et al. [134], the photoprotective effects of synthetic lycopene after 12 weeks of supplementation were examined, and significant increases in the lycopene serum and total skin carotenoid levels were detected. Studies of animals and humans suggested that green tea polyphenols are photoprotective and can be administered to prevent solar UVB light-induced skin disorders [137]. A review of the research reveals that polyphenols or other phytochemicals, such as green tea polyphenols, grape seed proanthocyanidins, resveratrol, silymarin, genistein, and others, exert substantial photoprotective effects against UV-induced skin inflammation, oxidative stress, DNA damage, and so forth.

Presently, we are exposed to various sources of radiation, both ionising and nonionising. The results of many studies indicate that the human body can cope with radiation-induced oxidative stress to a certain degree by consuming an appropriate antioxidant diet.

### 2.3. Pesticide-Induced Oxidative Stress and Protection against It

Pesticides have become an integral constituent of the ecosystem due to their widespread use, distribution, and the stability of some of the pesticides in the environment. Pesticide exposure may play a major role in increased oxidative stress of the organisms and may result in altered disease susceptibility. Bagchi et al. [145] demonstrated that pesticides induce the production of ROS and oxidative damage to tissues. de Liz Oliveira Cavalli [146] found that exposure to glyphosate causes oxidative stress and activates multiple stress-response pathways leading to Sertoli cell death in prepubertal rat testis. The role of oxidative stress in immune cell toxicity induced by the pesticides lindane, malathion, and permethrin was examined by Olgun and Misra [147]. Hassoun et al. [148] reported that chlordane produces oxidative tissue damage based on the levels of hepatic lipid peroxidation and DNA damage (Table 3). Bus et al. [149] reported that paraquat pulmonary toxicity results from the cyclic reduction and oxidation of paraquat. The results of a study performed by Pérez-Maldonado et al. [150] demonstrated the induction of apoptosis by DDT. Hassoun et al. [148] reported that lindane, DDT, chlordane, and endrin exposure resulted in significant increases in hepatic lipid peroxidation and DNA damage. Another study by Senft et al. [151] found out that dioxin increases mitochondrial respiration-dependent ROS production. On the other hand, Ciftci et al. [152] reported a protective effect of curcumin on the immune system of rats intoxicated with 2,3,7,8-tetrachlorodibenzo-p-dioxin. Additionally, Hung et al. [153] suggested that tea melanin might be a potential agent against the development of tetrachlorodibenzodioxin-induced oxidative stress. Gultekin et al. [154] examined the effects of melatonin and vitamins E and C on the reduction of chlorpyrifos-ethyl.

Another group of pesticides are polychlorinated biphenyls (PCBs), which also induce increased intracellular ROS production. Zhu et al. [155] indicated that different PCB compounds (Aroclor 1254, PCB153, and the 2-(4-chlorophenyl)-1,4-benzoquinone metabolite of PCB3) increase the steady-state levels of intracellular O_2_^•−^ and H_2_O_2_ in breast and prostate epithelial cells. Many antioxidants showed protection also against PCB-induced oxidative stress and damage. Ramadass et al. [156] tested the hypothesis that flavonoids modify PCB-mediated cytotoxicity and found that flavonoids inhibit PCB-induced oxidative stress. Zhu et al. [155] demonstrated that treatment with N-acetylcysteine significantly protected cells against PCB-mediated toxicity. Red ginseng, which displays a variety of biological and pharmacological activities, including antioxidant, anti-inflammatory, antimutagenic, and anticarcinogenic effects, was found to protect the body against oxidative stress/damage induced by PCB exposure [157]. Sridevi et al. [158] also reported that the effect of alpha-tocopherol against PCB-induced neurotoxicity resulted in decreased oxidative stress. Another study reported the synergistic effects of vitamins C and E against PCB- (Aroclor 1254) induced oxidative damage [159].

Dioxins and furans are byproducts of chemicals production. Dioxins may be released into the environment through the production of pesticides and other chlorinated substances. Both dioxins and furans are related to a variety of incineration reactions and the use of a variety of chemical products. Ciftci and coworkers reported that dioxin (2,3,7,8-tetrachlorodibenzo-p-dioxin; TCDD) causes an oxidative stress response in the rats liver. The subcellular sources and underlying mechanisms of dioxin-induced reactive oxygen species, however, are not well understood. TCDD increases the formation of thiobarbituric acid-reactive substances. It also causes a significant decline in the levels of glutathione, catalase, GSH-Px, and Cu-Zn superoxide dismutase in rats [160]. The impact of 2-furan-2-yl-1H-benzimidazole on vitamins A, E, C, and Se, malondialdehyde, and glutathione peroxidase levels on rats was analysed in a study by Karatas et al. [161]. The results showed that vitamins A, E, C, and Se levels were lower than the control groups, while serum MDA level and GSH-Px activity flexibly increased, depending on the injection days. The observed decreases in vitamins A, E, C, and Se levels in the blood might be causally related to the increased amount of ROS. The potential protective effect of quercetin on TCDD induced testicular damage in rats was studied by Ciftci et al. [160]. The results showed that exposure to TCDD induces testicular damage, and quercetin prevents TCDD-induced testicular damage in rats. Resveratrol's antioxidative effects were also investigated against in a study by Ishida et al. [162]. The results suggested that oral resveratrol is an attractive candidate for combating dioxin toxicity. Türkez et al. [163] analysed effects of propolis against TCDD induced hepatotoxicity in rats and found that propolis alleviate pathological effects and prevents the suppression of antioxidant enzymes in the livers.

It can be concluded that the stimulation of ROS production, the induction of lipid peroxidation and oxidative DNA and protein damage, and the disturbance of the total antioxidant capacity of the body are mechanisms of the toxicity induced by most pesticides, including organophosphates, bipyridyl herbicides, and organochlorines. Antioxidant nutrients and related bioactive compounds common in fruits and vegetables as well as food additives can protect against environmental exposure to pesticides-induced oxidative stress/damage (Table 3).

### 2.4. Household Chemical-Induced Oxidative Stress and Protection against It

The predominant use of industrial resins, such as urea-formaldehyde, phenol-formaldehyde, polyacetal, and melamine-formaldehyde resins, can be found in domestic environments in adhesives and binders for wood products, pulp products, paper products, plastics, synthetic fibres, and in textile finishing. Formaldehyde was demonstrated to exert increased oxidative stress formation (Table 4), primarily as lipid peroxidation, as found in a study performed by Chang and Xu [193]. Also in the case of household chemical-induced oxidative stress certain antioxidants showed protection. In a recent study, Köse et al. [194] reported that rose oil inhalation protects against formaldehyde-induced testicular damage in rats. Zararsiz et al. [195] demonstrated that exposure to formaldehyde increased the free radical levels in rats and that omega-3 fatty acids prevented this oxidative stress. The protective effect of melatonin against formaldehyde-induced renal oxidative damage in rats has also been reported [196].

Many studies have been performed on carbon tetrachloride because it is a well-known model of inducing chemical hepatic injury in mice. Also carbon tetrachloride exposure increases oxidative stress/damage in tested model organisms and carbon tetrachloride-induced damage has been reversed by many antioxidants examined. Thus, the antioxidant and hepatoprotective effects of many antioxidants and plant extracts against oxidative stress induced by carbon tetrachloride have been reported [198]. For example, chlorella-mediated protection against carbon tetrachloride-induced oxidative damage in rats was demonstrated in a study by Peng et al. [224]. Ozturk et al. [201] found that apricot (*Prunus armeniaca L*) feeding exerted beneficial effects. The potency of vitamin E to enhance the recovery from carbon tetrachloride-induced renal oxidative damage in mice was revealed in a study by Adaramoye [202]. The protective effects of* Curcuma longa *Linnwere reported by Lee et al. [205]. The protective effect of blackberry extract against oxidative stress in carbon tetrachloride-treated rats was reported by Cho et al. [207].

Chemicals found in common household and personal care goods are major sources of oxidant exposure that can lead to oxidative stress. Many antioxidants, such as melatonin, vitamin E, ascorbate, and extracts from various plants, for example, rose, green tea, and blackberry, were reported to decrease oxidative stress and/or damage in vivo and in vitro.

### 2.5. Disinfection Byproducts (DBP) and Other Water Born Pollutants

The beneficial role of water ingestion can be minimised due to the formation of disinfection byproducts. Chlorination and ozonation in the water treatment process are believed to produce various active oxygen species, which seem to participate in the reaction with fumic acid, pollutants, and bacteria (Table 4). Hypochlorous acid (HOCl) is formed when Cl_2_ is added to the water for disinfection purposes. This acid is highly reactive and is capable of oxidising many biological molecules. HOCl reacts with O_2_^−^ to give OH^•^ and with H_2_O_2_ to form singlet O_2_. HOCl affects endogenous enzymatic antioxidants and increases oxidative stress. For example, Hassoun and Ray [212] demonstrated the induction of oxidative stress and cellular death of drinking water disinfection byproducts. Similar observations were reported by Leustik et al. [214]. Studies suggest that Cl_2_ inhalation damages both airway and alveolar epithelial tissues and that these damaging effects were ameliorated by the prophylactic administration of low molecular-weight antioxidants. Trolox was reported to be protective against oxidative injury induced by HOCl to Ca-ATPase in the sarcoplasmic reticulum of skeletal muscle [220]. Ascorbic acid might also play a protective role (Table 4), especially in individuals consuming supplements containing this vitamin. Also thioallyl and S-allylcysteine (both are garlic-derived compounds), melatonine, glutathione, glutathione disulfide, S-methylglutathione, lipoic acid, and dihydrolipoic acid were reported to protect against hypochlorous acid and peroxynitrite-induced damage [217–219, 222]).

Additionally, the following plant extracts display a protective effect against HOCl-induced oxidative damage:* Agaricus campestris*,* Cynara cardunculus*,* Thymus pulegioides*, and* Vicia faba* [223]. When resolving the problem of DBP, first the cause of their formation should be assessed with different engineering approaches DBP, for example, by moving the point of chlorination downstream in the treatment train, reducing the natural organic matter precursor concentration, replacing prechlorination by peroxidation, and so forth.

The use of antioxidants as compounds which ameliorate DBP-induced toxicity should be just the last alternative when all other approaches deal with the DBP formation in the drinking water fail.

Researches in the past two decades have pointed out that redox active metals like iron (Fe), copper (Cu), chromium (Cr), cobalt (Co), and other metals present in water possess the ability to produce ROS such as superoxide anion radical and nitric oxide. Disruption of metal ion homeostasis may lead to oxidative stress, a state where increased formation of reactive oxygen species overwhelms body antioxidant protection and subsequently induces DNA damage, lipid peroxidation, protein modification, and other effects [225]. Pollutants in water like heavy metals As, Cd, Cu, Fe, Pb, and Zn can cause oxidative stress in fish [226]. On other hand Yang and coworkers [227] reports that water spinach containing chlorophyll and lycopene have potential to reduce cytotoxicity and oxidative stress in liver induced by heavy metals. Besides heavy metals also pesticides in water can represent sources of oxidative stress. Atrazine and chlorpyrifos are the most common pesticides found in freshwater ecosystems throughout the world. Xing et al. [228] investigated the oxidative stress responses in the liver of common carp after exposure to atrazine and chlorpyrifos and found that exposure or their mixture could induce decrease in antioxidant enzyme activities and increase in MDA content in a dose-dependent manner. Eroğlu et al. [229] reported organophosphate pesticides produce oxidative stress due to the generation of free radicals, which alter the antioxidant defence system in erythrocytes and that vitamins C and E can act as protective role.

### 2.6. The Role of Oxidative Stress in Noise-Induced Hearing Damage

Noise is a disturbing and unwanted sound. Exposure to noise causes many health problems such as hearing loss, sleep disturbance, and impairs performance as well as effecting cognitive performance. It also increases aggression and reduces the processing of social cues seen as irrelevant to task performance, as well as leading to coronary heart disease, hypertension, higher blood pressure, increased mortality risk, serious psychological effects, headache, anxiety, and nausea ([230] and references within). Prolonged exposure to noise can also cause oxidative stress in the cochlea which results in the loss (via apoptotic pathways) of the outer hair cells of the organ of Corti. Increased noise exposure results in increased levels of reactive oxygen species formation that play a significant role in noise-induced hair cell death [231]. Acute as well as long-term exposure to noise can produce excessive free radicals alter endogenous antioxidative enzymes as superoxide dismutase, catalase, and glutathione peroxidase [232, 233].

In a study by Demirel et al. [230] the effect of noise on oxidative stress parameters in rats was analyzed by measuring malondialdehyde, nitric oxide levels, and glutathione peroxidase activity. The results showed an elevation in MDA level, an indicator of lipid peroxidation, as well as NO level and GSH-Px activity through noise exposure, suggesting that the presence of oxidative stress may have led to various degrees of damages in the cells. Additionally, increases in oxidative stress parameters, such as MDA level, and decreases in CAT and SOD activities in textile workers exposed to elevated levels of noise supports the hypothesis that noise causes oxidative stress [234]. It seems that noise might cause damage not only in the ears but also across the entire body, leading to oxidative stress [230]. In a study by van Campen et al. [235], the time course of ROS damage following exposure was assessed. Based upon oxidative DNA damage present in the cochlea following intense noise, the researchers postulate that the first 8 h following exposure might be a critical period for antioxidant treatment. Thus, the ROS quenching properties of antioxidants and medicinal plants are attracting more and more research to counteract noise-induced oxidative stress. Manikandan and Devi [232] investigated the antioxidant property of alpha-asarone against noise stress induced changes in different regions of the rat brain and their data proved that the antioxidant property of alpha-asarone acts against noise stress induced damage. The aim of a study performed by Manikandan et al. [233] was to evaluate the protective effect of both ethyl acetate and methanolic extract of* Acorus calamus* against noise stress induced changes in the rat brain. Both the ethyl acetate and methanolic extract of* Acorus calamus* protected most of the changes in the rat brain induced by noise stress. N-acetyl-cysteine also offered protection against noise-induced hearing loss in the Sprague Dawley rat [236]. The study by Ewert et al. [237] determined if administration of a combination of antioxidants 2,4-disulfonyl *α*-phenyl tertiary butyl nitrone (HPN-07) and N-acetylcysteine could reduce both temporary and permanent hearing loss. The results showed that a combination of antioxidants HPN-07 and NAC can both enhance the temporary threshold shift recovery and prevent permanent threshold shift by reducing damage to the mechanical and neural components of the auditory system when administered shortly after blast exposure. Additionally, arboxy alkyl esters (esters of quinic acid found in fruits and vegetables) have been shown to improve DNA repair capacity of spiral ganglion neurons in response to noise stress [238].

The problem of oxidative stress in the production of hearing loss is even worse when the synergistic effects takes place since a broad range of environmental and occupational contaminants can interact with noise to enhance noise-induced hearing loss, for example, through carbon monoxide and by acrylonitrile [239].

### 2.7. Adverse or Insignificant Effects of Antioxidant Treatment after Exposure to Environmental Pollutants

Administration of antioxidants in cases of environmentally induced oxidative stress does not always demonstrate protection (Table 5). Hackney et al. [240] analysed whether vitamin E supplementation protected against O_3_ exposure and found no significant differences between the vitamin E- and placebo-treated groups. Another study demonstrated that in a high-risk group, such as smokers, high doses of beta-carotene increased the rate of lung cancer [241]. Additionally, the results of large, controlled trials of an intervention of beta-carotene supplementation did not support the detected beneficial associations or a role for supplemental beta-carotene in lung cancer prevention; instead, they provided striking evidence for its adverse effects among smokers [242]. McArdle et al. [118] investigated the effects of oral vitamin E and beta-carotene supplementation on ultraviolet radiation-induced oxidative stress to the human skin. The results revealed that vitamin E or beta-carotene supplementation displayed no effect on the sensitivity of the skin to UVR. A study by Stahl et al. [122] was performed in which the antioxidant effect of carotenoids and tocopherols was investigated based on their ability to scavenge ROS generated during photooxidative stress. The antioxidants used in this study provided protection against erythema in humans and may be useful for diminishing the sensitivity to ultraviolet light (Table 5).

Iron and copper have been reported to aggravate the toxicity of paraquat in* E. coli*. Treatment with ferrous iron in a study by Korbashi et al. [248] led to an enhancement of bacterial killing by paraquat, whereas treatment with chelating agents, such as nitrilotriacetate and desferrioxamine, markedly reduced, up to complete abolishment, the toxic effects. Some compounds contribute to the antioxidant defence by chelating transition metals and preventing them from catalysing the production of free radicals in the cell. Metal-chelating antioxidants, such as transferrin, albumin, and ceruloplasmin, ameliorate radical production by inhibiting the Fenton reaction, which is catalysed by copper or iron. Latchoumycandane and Mathur [250] investigated whether treatment with vitamin E protects the rat testis against oxidative stress induced by tetrachlorodibenzodioxin and revealed that the activities of antioxidant enzymes and the levels of hydrogen peroxide and lipid peroxidation did not change in the animals coadministered tetrachlorodibenzodioxin and vitamin E. Although several studies have demonstrated the protective effect of antioxidant administration against oxidative stress, it is important to note that not all antioxidants exert health benefits.

### 2.8. What Could Be the Reason? 

The inappropriate use of dietary supplements may lead to “antioxidative stress.” Detailed description of the negative effects of antioxidants can be found in publications by Poljsak et al., [253], Poljsak and Milisav [254], and references therein. Briefly, the intake of only one antioxidant may alter the complex system of endogenous antioxidative defence of cells or alter the cell apoptosis pathways [255]. The beneficial physiological cellular use of ROS is being demonstrated in different fields, including intracellular signalling and redox regulation and synthetic antioxidants cannot distinguish among the radicals that have a beneficial role and those that cause oxidative damage to biomolecules. If administration of antioxidant supplements decreases total ROS/RNS formation, it may also interfere with the immune system to fight bacteria and essential defensive mechanisms for removal of damaged cells, including those that are precancerous and cancerous [256]. When large amounts of antioxidant nutrients are taken, they can also act as prooxidants by increasing oxidative stress [257, 258]. None of the major clinical studies using mortality or morbidity as an end point has found positive effects of antioxidant, such as vitamin C, vitamin E, or *β*-carotene, supplementation. Some recent studies demonstrated that antioxidant therapy displays no effect and can even increase mortality (The Alpha-Tocopherol, Beta-Carotene Cancer Prevention Study Group, 1994; [259–261], Heart Protection Study Collaborative Group, 2002; Age-Related Eye Disease Study Research Group, 2001). On the other hand, antioxidant supplements do appear to be effective in lowering an individual's oxidative stress if his/her initial oxidative stress is above normal or above his/her set point of regulation [262, 263]. Thus, the antioxidant supplements may help the organism to correct the elevated levels of oxidative stress when it cannot be controlled by the endogenous antioxidants.

## 3. Conclusions

There is substantial evidence that environmental pollution increases oxidative stress [264] and that dietary antioxidant supplementation and/or increased ingestion of fruit and vegetable may play a role in neutralising or buffering the effects of pollutants that display oxidising properties. In vitro and in vivo studies suggest that antioxidant nutrients and related bioactive compounds common in fruits and vegetables can protect against environmental toxic insults. It is important to emphasise that antioxidants as dietary supplements can provide protection against ROS-induced damage under conditions of elevated oxidative stress to the organism. It could be postulated that antioxidants would be therapeutically effective under circumstances of elevated oxidative stress or in aged mammals exposed to a stressor that generates exacerbated oxidative injury. Evidence is presented demonstrating that synthetic antioxidant supplements cannot provide appropriate or complete protection against oxidative stress and damage under “normal” conditions and that the administration of antioxidants to prevent disease or the aging process is controversial under conditions of “normal” oxidative stress. Many clinical trials in which individuals received one or more synthetic antioxidants failed to detect beneficial effects (reviewed in [253]). Thus, the results of clinical trials of exogenous antioxidant intake are conflicting and contradictory. These findings indicate that other compounds in fruits and vegetables (possibly flavonoids) or a complex combination of compounds may contribute to the improvement in cardiovascular health and the decrease in cancer incidence detected among individuals who consume more of these foods [265, 266].

It must be understood that the use of synthetic vitamin supplements is not an alternative to regular consumption of fruits and vegetables. Cutler explains that most humans maintain stable levels of oxidative stress, and no matter how much additional antioxidant that individuals consume in their diet, no further decrease in oxidative stress occurs. However, antioxidant supplements do appear to be effective in lowering an individual's oxidative stress if his/her initial oxidative stress level is above normal or above his/her stably regulated level [262, 263]. Thus, antioxidant supplements may only provide a benefit to an organism if it was necessary to correct a high level of oxidative stress that could not be controlled by endogenous antioxidants. All of this evidence indicates the need to determine an individual's oxidative stress level prior to the initiation of antioxidant supplement therapy. Both, the ROS/RNS formation and the antioxidative defense potential should be measured in a person in order to determine his/her oxidative stress status. Multiple methods of oxidative stress measurement are available today, each with their own advantages and disadvantages (reviewed in [253]).

In the end it should be stressed that more research should be performed to strengthen the evidence for dietary supplements as modulators of the adverse effects caused by increased exposure to environmental pollution.

## Figures and Tables

**Table 1:** Studies demonstrating increased oxidative stress/damage due to air pollutant exposure and the protective effects of antioxidants.

Air pollutant	Increased oxidative stress markers	Study	Antioxidants exerting a protective effect	Study
Oxygen (O_2_)	Superoxide and hydrogen peroxide generation	Floyd (1995) [8]	Catalases, glutathione peroxidases, and peroxiredoxins	Nordberg and Arnér (2001) [9]
	Hydroxyl radical (OH^*∙*^)	Forman and Boveris (1982) [10] Keyer and Imlay (1996) [11] Hutchinson (1985) [12] Ames (1983) [13]		
	Oxidative DNA lesions	Friedberg et al. (1995) [14] Speakman et al. (2003) [15] Shackelford et al. (1999) [16]		

Ozone (O_3_)	Antioxidant depletion	Mudway et al. (1996) [17] Pryor (1992) [18] Cross et al. (2002) [3]	Vitamins C and E and beta-carotene	Grievink et al. (1999; 1997) [19, 20] Samet et al. (2001) [21] Menzel (1994) [22] Romieu et al. (2002) [23] Romieu et al. (1998) [24]
	Protein oxidation	Kelly and Mudway (2003) [5]		Grievink et al. (2000) [25]
	Membrane oxidation	Ballinger et al. (2005) [26]		
	Inflammation	Menzel (1994) [22]		

Sulphur dioxide (SO_2_)	TBARS	Meng et al. (2003) [27] Meng and Bai (2004) [28]	Vitamin E	Ergonul et al. (2007) [29] Etlik et al. (1997) [30]
		Zhao et al. (2009) [31]	Vitamin C	
	Depletion of endogenous antioxidants	Etlik et al. (1997, 1995) [30, 32] Zhao et al. (2008) [33]	Salicylic acid and vitamin C	Zhao et al. (2009) [31]
	Malondialdehyde	Wu and Meng (2003) [34]	GSH	Langley-Evans et al. (1996) [35]
	Change in the glutathione redox system		Sea buckthorn seed oil	Wu and Meng (2003) [34]

Cigarette smoke	Decreased antioxidant capacity	Midgette et al. (1993) [36] Banerjee et al. (1998) [37] Bloomer (2007) [38] Aycicek et al. (2005) [39] Tsuchiya et al. (2002) [40] Zhou et al. (2000) [41]	Vitamin C	Banerjee et al. (2008) [42] Mayne and Cartmel (1999) [43]
			Cruciferous vegetables and green tea ((−)-epigallocatechin gallate (EGCG) and caffeine)	Chung et al. (1993) [44] Xu et al. (1992) [45]
	Lipid peroxidation	Banerjee et al. (1998) [37] Jha et al. (2007) [46]	Black tea	Chung (1999) [47]
	Oxidation of purines	Jha et al. (2007) [46]		
	8-OH-dGuo	Xu et al. (1992) [45]		
	Decreased antioxidant vitamin activities	Zhou et al. (1997) [48] Dietrich et al. (2003) [49] Chávez et al. (2007) [50] Bloomer (2007) [38]	Tomato-based juice, vitamin E, and beta-carotene	Mayne and Cartmel (1999) [43]
	Protein damage and inflammation	Banerjee et al. (2008) [42]		
	Malondialdehyde (MDA)	Chávez et al. (2007) [50] Polidori et al. (2003) [51]		

Nitrogen oxides (NO_*x*_)	Aldehydes, hydrogen peroxide, and reactive oxygen intermediates	Pryor and Church (1991) [52] Last et al. (1994) [53] Kienast et al. (1994) [54]	Vitamin E	Guth and Mavis (1986) [55] Sevanian et al. (1982) [56]
	Depletion of antioxidants	Kelly and Tetley (1997) [57] Kelly et al. (1996) [58]	Vitamin C	Rietjens et al. (1986) [59] Mohsenin (1987) [60]
	Lipid peroxidation	Sevanian et al. (1982) [56] Khopde et al. (1998) [61]	Lycopene	Böhm et al. (2001) [62]

Particulate matter (PM)	Direct generation of ROS	González-Flecha (2004) [63]	N-acetylcysteine and deferoxamine	Pinho et al. (2005) [64]
	Proinflammatory mediators released from PM-stimulated macrophages	González-Flecha (2004) [63]		
	Oxidative DNA damage	González-Flecha (2004) [63] Aganasur et al. (2001) [65]		
	Inhibitory effects on oxidative stress-related enzymes	Hatzis et al. (2006) [66]		
	Thiobarbituric acid reactive substances, protein carbonyls	Possamai et al. (2010) [67]	vitamins C and E	Possamai et al. (2010) [67]

Asbestos	ROS formation (oxygen free radicals)	Kamp et al. (1992) [68] Walker et al. (1992) [69]	Green tea extract	Luo et al. (1995) [70]
	Hydrogen peroxide, hydroxyl radical, and superoxide anion	Lewczuk and Owczarek (1992) [71]	SOD	Fattman et al. (2006) [72]
	Activation of phagocytic cells	Kamp et al. (1992) [68] Hei et al. (2006) [73] Walker et al. (1992) [69]		
	Increased 8-isoprostane	Pelclová et al. (2008) [74]		
	8-Hydroxy-2′-deoxyguanosine	Marczynski et al. (2000) [75]		

**Table 2:** Studies demonstrating increased oxidative stress/damage due to ionising and nonionising radiation exposure and the protective effects of antioxidants.

Radiation	Increased oxidative stress markers	Study	Antioxidants exerting a protective effect	Study
Ionising radiation	8-OHdG	Sperati et al. (1999) [84]	Ebselen	Tak and Park (2009) [85]
	ROS: superoxide (O_2_^∙−^) and the hydroxyl radical (OH^*∙*^)		N-acetylcysteine	Kilciksiz et al. (2008, 2011) [86, 87]
	DNA damage and lipid membrane damage		Quercetin and the ethanolic extract of propolis	Benković et al. (2009) [88]
			L-selenomethionine, vitamin C, vitamin E, succinate, the combination of alpha-lipoic acid and N-acetyl cysteine	Wambi et al. (2009) [97] Andrade et al. (2011) [98]
			Curcumin	Jagetia (2007) [89]
			Sesamol	G. G. Nair and C. K. K. Nair (2010) [99]
			Melatonin vitamin E	El-Missiry et al. (2007) [100] Karbownik and Reiter (2000) [101]
			Lycopene	Nair et al. (2003) [102] Noaman et al. (2002) [103] Berbée et al., (2009) [104]
			Green tea polyphenols	Srinivasan et al. (2009) [105] Hu et al. (2011) [106]

Nonionising radiation	ROS production	Kovacic and Somanathan (2010) [90]	Alpha-tocopherol	Wolf et al. (2005) [107]
		Simkó and Mattsson (2004) [91]	N-acetyl-L-cysteine and epigallocatechin-3-gallate	G$\ddot{\text{u}}$ler et al. (2008) [108] Ozgur et al. (2010) [109]
		Musaev et al. (2004) [92]		
	Enhanced lipid peroxidation and altered antioxidant defence systems	Simkó (2007) [110]	Melatonin and caffeic acid phenethyl ester	Ozguner et al. (2006) [93] Jajte et al. (2001) [94] Reiter (1994) [111]
	DNA damage	Jajte et al. (2001) [94]	*Ginkgo biloba *	Ilhan et al. (2004) [95]
			L-carnitine and selenium	Naziroğlu and Gümral (2009) [112]
			Vitamins E and C	Guney et al., (2007) [96]

UVR	Generation of oxidants via photodynamic action (e.g., H_2_O_2_, singlet oxygen, peroxy radicals, superoxide anion, and hydroxyl radicals)	Peak et al. (1988) [113] Beehler et al. (1992) [114] Berton et al. (1997) [115] Li et al. (1996) [116] Masaki (2010) [117]	Vitamin C	McArdle et al. (2002) [118] Humbert et al. (2003) [119]
			Tocopherol	Ritter et al. (1997) [120] Packer et al. (2001) [121] Stahl et al. (2000) [122]
	Photochemical damage to cellular DNA	J. H. Kligman and A. M. Kligman (1986) [123]	Vitamin A, beta-carotene, and other carotenoids	Stahl et al. (2006) [124] Sies and Stahl (2004) [125] Cho et al. (2010) [126] Stahl et al. (2000) [122]
	Photoaging	McArdle et al. (2002) [118] Shindo et al. (1993) [127] Packer and Valacchi (2002) [128]		
	Depletion of antioxidants	Thiele (2001) [129] Ribaya-Mercado et al. (1995) [130]	Carotenoids, beta-carotene, mixture of lutein and lycopene	Heinrich et al. (2003) [131] Lee et al. (2000) [132] Stahl et al. (1998) [133]
			Lycopene	Stahl et al. (1998) [133] Aust et al. (2005) [134] Yeh et al. (2005) [135]
			Resveratrol	Afaq and Mukhtar (2002) [136]
			Green tea polyphenols and other flavonoids	Katiyar et al. (2000) [137] Katiyar (2003) [138] Katiyar et al. (2010) [139] Lu, et al. (2008) [140] Singh and Agarwal (2002) [141] Bonina et al. (1996) [142] Wei et al. (1995) [143]
			Pycnogenol	Saliou et al. (2001) [144]

**Table 3:** Studies demonstrating increased oxidative stress/damage due to pesticide exposure and the protective effects of antioxidants.

Pesticide	Increased oxidative stress markers	Study	Antioxidants exerting a protective effect	Study
General	ROS	Bagchi et al. (1995) [145] Song et al. (2007) [164] Olgun and Misra (2006) [147]		
	DNA damage	Bagchi et al. (1995, 1996) [145, 165] Kisby et al. (2009) [166]		
	Alterations in antioxidant enzymes and the glutathione redox system	Yi et al. (2007) [167]		
	Increased level of malondialdehyde Lipid peroxidation	Kesavachandran et al. (2006) [168] Hassoun et al. (1993) [148]		

Bipyridylium herbicides (paraquat, diquat and difenzoquat)	Production of superoxide anions and singlet oxygen	Bus et al. (1976) [149]	Selenium	Combs and Peterson (1983) [169] Glass et al. (1985) [170] Cheng et al. (1998) [171]
	Lipid peroxidation (peroxidation of cellular membranes)	Bus et al. (1976) [149]		
	Alterations in antioxidant enzymes and the glutathione redox system	Takizawa et al. (2007) [172]	Glutathione reductase and superoxide dismutase	Aono et al. (1995) [173]

Organo-phosphate insecticides	Lipid peroxidation	Gultekin et al. (2001) [154] Akturk et al. (2006) [174] Hassoun et al. (1993) [148]	Melatonin, vitamin C and vitamin E	Gultekin et al. (2001) [154]
	Decreased antioxidant defence	Gultekin et al. (2001) [154] Verma et al. (2007) [175] Akturk et al. (2006) [174]	Vitamins A, E, and C	Verma et al. (2007) [175] Altunas et al. (2002) [176] Akturk et al. (2006) [174]
	Increased ROS production	Bagchi et al. (1995) [145]		
	DNA damage	Bagchi et al. (1995) [145]	Zinc	Goel et al. (2005) [177]

Aldrin and dieldrin	ROS production	Stevenson et al. (1999) [178]	Alpha-tocopherol and ascorbic acid	Bachowski et al. (1998) [179] Stevenson et al. (1995) [180]
	Depletion of the antioxidant defence	Klaunig et al. (1995) [181]		
	Lipid peroxidation	Bachowski et al. (1998) [179] Klaunig et al. (1995) [181]		
	DNA damage	Klaunig et al. (1995) [181]		

DDT	ROS production	Pérez-Maldonado et al. (2005) [150]	N-acetyl-L-cysteine	Pérez-Maldonado et al. (2005) [150]
	Lipid peroxidation	Hassoun et al. (1993) [148]		
	DNA damage	Hassoun et al. (1993) [148]		

Polychlorinated dibenzo-para-dioxins (dioxins) and polychlorinated dibenzo furans (furans)	Depletion of the antioxidant defence	Ciftci et al. (2011) [160] Stohs (1990) [182] Karatas et al. (2008) [161]	Quercetin	Ciftci et al. (2011) [160]
	Lipid peroxidation	Stohs (1990) [182] Karatas et al. (2008) [161]	Curcumin, *β*-myrcene and 1,8-cineole	Ciftci et al. (2011) [160] Ciftci et al. (2010) [152]
			Resveratrol	Ishida et al. (2009) [162]
			Tea melanin	Hung et al. (2006) [153]
	TBARS	Ciftci et al. (2011) [160]	Vitamin A and vitamin E	Alsharif and Hassoun (2004) [183]
	Increased ROS production	Senft et al. (2002) [151]		
	8-OHdG	Wen et al. (2008) [184]		
	DNA damage	Stohs (1990) [182]		

Polychlorinated biphenyls (PCBs)	ROS production (superoxide and hydrogen peroxide)	Song et al. (2008) [185] Zhu et al. (2009) [155]	Dietary flavonoids (epigallocatechin-3-gallate (EGCG) and quercetin)	Ramadass et al. (2003) [156]
	Imbalance in the antioxidant status	Zhu et al. (2009) [155] Shimizu et al. (2007b) [186]	N-acetylcysteine (NAC) and the combination of polyethylene glycol (PEG)-conjugated Cu/Zn-SOD and PEG-conjugated catalase	Zhu et al. (2009) [155]
	Lipid peroxidation	Shimizu et al., (2007a) [187]	Red ginseng	Park et al. (2010) [157]
			Alpha-tocopherol	Banudevi et al. (2006) [188] Sridevi et al. (2007) [158]
			Alpha-tocopherol and ascorbic acid	Krishnamoorthy et al. (2007) [189] Murugesan et al. (2005) [159] Zhou and Zhang (2005) [190]
			Melatonin	Venkataraman et al. (2008) [191]
			Lycopene	Elumalai et al. (2009) [192]

**Table 4:** Studies demonstrating increased oxidative stress/damage due to exposure to selected toxic compounds and the protective effects of antioxidants.

Other	Increased oxidative stress markers	Study	Antioxidants exerting a protective effect	Study
Formaldehyde	Lipid peroxidation	Chang and Xu (2006) [193]	Rose oil	Köse et al. (2011) [194]
	Imbalance in antioxidant status	Chang and Xu (2006) [193]	Melatonin	Zararsiz et al. (2007) [196]

Carbon tetrachloride (CCl_4_)	Increased ROS production	Brent and Rumack (1993) [197]	Electrolysed reduced water	Tsai et al. (2009) [198]
	Lipid peroxidation	Morrow et al. (1992) [199] Basu (2003) [200]	Apricot (*Prunus armeniaca *L.)	Ozturk et al. (2009) [201]
			Vitamin E	Adaramoye (2009) [202]
			Megahydrate silica hydride Lutein	Hsu et al. (2010) [203] Sindhu et al. (2010) [204]
			Curcuma longa	Lee et al. (2010) [205]
			Acetyl-L-carnitine	Annadurai et al. (2011) [206]
			Blackberry extract	Cho et al., (2011) [207]
			Capsaicin	Hassan et al. (2012) [208]
			Propolis	Bhadauria (2012) [209]
			Melatonin and pinoline	Aranda et al. (2010) [210]
			Black and green tea	Almurshed (2006) [211]

Water disinfection byproducts	ROS production (OH^*∙*^, H_2_O_2_, and singlet O_2_)	Hassoun and Ray (2003) [212]	Ascorbate, glutathione, and urate	Yadav et al. (2010) [213]
			Ascorbate and desferal N-acetyl-cysteine	Yadav et al. (2010) [213] Leustik et al. (2008) [214]
			Ascorbate and deferoxamine	Zarogiannis et al. (2011) [215]
			Green tea catechins	Kawai et al. (2008) [216]
			Melatonin	Tan et al. (2000) [217]
			S-allylcysteine	Medina-Campos et al. (2007) [218]
			Thioallyl compounds from garlic	Argüello-García et al. (2010) [219]
			Trolox	Strosova et al. (2009) [220]
			Vitamin C	Carr et al. (2000) [221]
			Glutathione	Rezk et al. (2004) [222]
			Many other local Mediterranean plant foods	Schaffer et al. (2004) [223]

**Table 5:** Studies demonstrating adverse or insignificant effects of antioxidant treatment after exposure to environmental pollutants.

Environmental pollutant	Antioxidants displaying adverse or insignificant effects	Study
Ozone (O_3_)	Vitamin E	Hackney et al. (1981) [240]

Cigarette smoke	Beta-carotene (synthetic)	Ruano-Ravina et al. (2006) [241] Albanes (1999) [242] Neuhouser et al. (2003) [243]

Asbestos	Beta-carotene	van Helden et al. (2009) [244]

UVR	Beta-carotene	Stahl et al. (2006) [124]
	Vitamin E and beta-carotene (no protective effect)	McArdle et al. (2004) [245] Wolf et al. (1988) [246] Garmyn et al. (1995) [247]

Bipyridinium herbicides (paraquat, diquat and difenzoquat)	Iron and copper	Korbashi et al. (1986) [248] Kohen and Chevion (1985) [249]

Polychlorinated dibenzo-para-dioxins (dioxins) and polychlorinated dibenzofurans (furans)	Vitamin E (no protective effect)	Latchoumycandane and Mathur (2002) [250]

No extraexposure to environmental pollutants	Green tea catechins (enhanced colon carcinogenesis in rats)	Furukawa et al. (2003) [251]
	Melatonin (may exhibit carcinogenic potential)	Sakano et al. (2004) [252]
